# Mimicking Protein–Protein Interactions through Peptide–Peptide Interactions: HIV-1 gp120 and CXCR4

**DOI:** 10.3389/fimmu.2013.00257

**Published:** 2013-09-03

**Authors:** Andrea Groß, Kalle Möbius, Christina Haußner, Norbert Donhauser, Barbara Schmidt, Jutta Eichler

**Affiliations:** ^1^Department of Chemistry and Pharmacy, University of Erlangen-Nuremberg, Erlangen, Germany; ^2^Institute of Clinical and Molecular Virology, University of Erlangen-Nuremberg, Erlangen, Germany; ^3^Institute of Medical Microbiology and Hygiene, University of Regensburg, Regensburg, Germany

**Keywords:** peptide, protein–protein interaction, HIV-1, gp120, coreceptor, CXCR4, V3 loop

## Abstract

We have recently designed a soluble synthetic peptide that functionally mimics the HIV-1 coreceptor CXCR4, which is a chemokine receptor that belongs to the family of seven-transmembrane GPCRs. This CXCR4 mimetic peptide, termed CX4-M1, presents the three extracellular loops (ECLs) of the receptor. In binding assays involving recombinant proteins, as well as in cellular infection assays, CX4-M1 was found to selectively recognize gp120 from HIV-1 strains that use CXCR4 for cell entry (X4 tropic HIV-1). Furthermore, anti-HIV-1 antibodies modulate this interaction in a molecular mechanism related to that of their impact on the gp120–CXCR4 interaction. We could now show that the selectivity of CX4-M1 pertains not only to gp120 from X4 tropic HIV-1, but also to synthetic peptides presenting the V3 loops of these gp120 proteins. The V3 loop is thought to be an essential part of the coreceptor binding site of gp120 that contacts the second ECL of the coreceptor. We were able to experimentally confirm this notion in binding assays using substitution analogs of CX4-M1 and the V3 loop peptides, respectively, as well as in cellular infection assays. These results indicate that interactions of the HIV-1 Env with coreceptors can be mimicked by synthetic peptides, which may be useful to explore these interactions at the molecular level in more detail.

## Introduction

Essentially all biological processes are initiated by specific interactions between proteins and their ligands. The design and generation of molecules capable of mimicking the binding and/or functional sites of proteins, represents a strategy for the exploration and modulation of protein function through controlled interference with the underlying binding events. In addition to their basic significance, such protein mimetics are also useful tools for a range of biomedical applications, in particular the inhibition of protein–protein interactions.

In general, synthetic peptides can be considered adequate tools for the mimicry of specific protein sites, since they can be generated as exact copies of protein fragments, as well as in diverse chemical modifications, which includes the incorporation of a large range of non-proteinogenic amino acids, as well as the modification of the peptide backbone (1). Apart from extending the chemical and structural diversity presented by peptides, such modifications also increase the proteolytic stability of the molecules, enhancing their potential as drug candidates.

Entry of the human immunodeficiency virus (HIV-1), the causative agent of the acquired immunodeficiency syndrome (AIDS), into its host cell, as well as its replication, is mediated by a range of specific interactions between viral and host cell proteins (2–4). Peptides mimicking the binding sites of the involved proteins are not only valuable tools to explore the respective interactions at the molecular level, but also candidates for therapeutic intervention through specific inhibition of these interactions (5). HIV-1 enters its host cell by using its envelope (Env), which is composed of the two glycoproteins gp120 and gp41. Env is located on the virus surface, where it forms trimeric spikes (6, 7). While gp120 is important for the attachment of the virus to the cellular receptor CD4, as well as coreceptors CXCR4 and CCR5, respectively, gp41 mediates fusion of the viral and cellular membranes. Consequently, peptides presenting fragments of Env proteins that are involved in virus-cell contact and fusion, are promising candidates for the inhibition of HIV-1 cell entry (8). In fact, the first and so far only HIV-1 fusion inhibitor approved for clinical use is a 36-mer peptide derived from gp41 (9, 10). The coreceptor binding site of Env is located in the bridging sheet and V3 loop of gp120 (11). This binding site, however, is properly exposed, i.e., accessible for the coreceptor, only upon prior contact of Env with CD4, which induces a conformational re-arrangement of gp120 (6, 7, 12, 13). According to their coreceptor usage (CXCR4 or CCR5), HIV-1 strains are classified as X4- and R5-tropic, respectively (14).

We have recently extended the scope of using scaffolded and assembled peptides for the synthetic mimicry of large, discontinuous protein binding sites, to extracellular domains of transmembrane proteins, i.e., the HIV-1 coreceptor CXCR4. The functionality of this CXCR4 mimetic peptide was demonstrated by its ability to discriminate between gp120 from X4- and R5-tropic HIV-1 in binding assays involving recombinant proteins, as well as in cellular infection assays (15).

**Figure 1:**
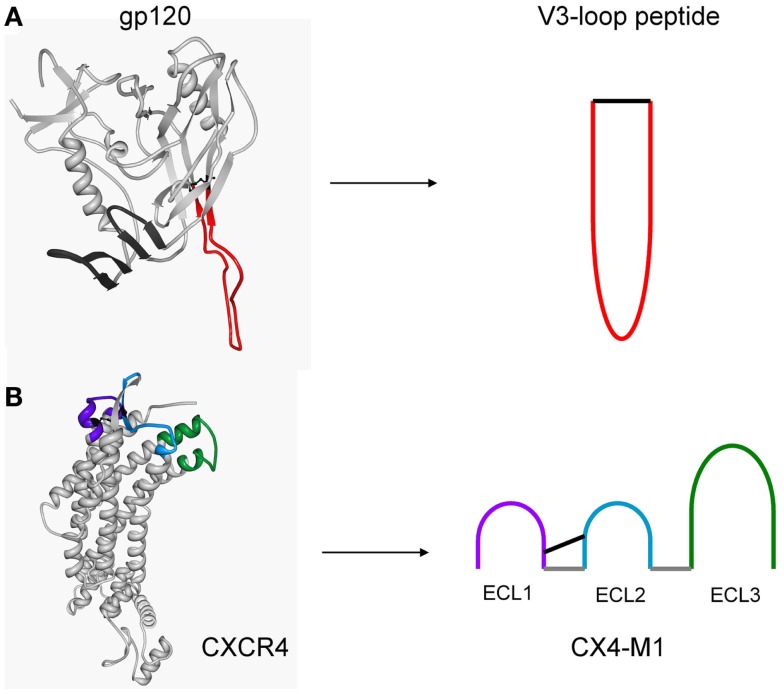
**Structure-based design of peptides presenting the V3 loop of gp120 (A), and the three extracellular loops of CXCR4 (CX4-M1) (B)**. Peptides were designed based on crystal structures of a gp120–CD4 complex (pdb: 2b4c) and CXCR4 (pdb: 3odu), respectively.

In this study, we aimed at exploring, using the gp120–CXCR4 interaction as a model, the scope and limitations of mimicking protein–protein interactions not only through protein–peptide, but also through peptide–peptide interactions, by means of mimicking the binding sites of both proteins through peptides. The larger degree of flexibility of linear, unfolded peptides, as compared to folded proteins, is generally thought to limit their potential for high-affinity interactions, since the reduction of conformational entropy is a major barrier that has to be overcome in protein binding (16). It has been shown, however, that intrinsically unfolded peptides can adopt defined conformations once they are bound to their target protein (17). Here, we asked the question if a protein–protein interaction can be mimicked by peptides presenting the binding sites of the proteins for each other. Specifically, we set out to generate peptides that mimic the V3 loops of various HIV-1 strains, and to assay their ability to specifically bind to the CXCR4 mimetic peptide CX4-M1 (Figure 1).

## Materials and Methods

### Materials

Gp120_IIIB_ was obtained from ImmunoDiagnostics, gp120_MN_, gp120_ADA_, and gp120_HxBc2_ from Immune Technology, sCD4 from Sino Biological, and mAb 447-52D from Polymun. MAb 3869 and gp120_BAL_ were obtained through the NIH AIDS Research and Reference Reagent Program. Fmoc-amino acids were obtained from Iris Biotech.

### Peptide synthesis

Peptides (see Tables 3 and 5 for sequences) were synthesized as C-terminal amides by Fmoc/t-Bu-based solid-phase synthesis on 100 mg TentaGel S RAM resin (0.23 mmol/g) using an automated multiple peptide synthesizer (SYRO from MultiSynTech). In a standard coupling cycle, five eq. of Fmoc-amino acid/DIC/HOBt in DMF were coupled twice for 60 min, followed by a capping step using a mixture of acetic anhydride/pyridine/DMF (1:2:3; 30 min). The biotin moiety of CX4-M1 and its variants was introduced by coupling three eq. of Fmoc-Lys(biotin)/DIC/HOBt in DMF over night. The Fmoc group was removed using 20% piperidine/DMF (20 min). The N-terminal amino groups were acetylated (CX4-M1 and its variants), fluoresceinylated (all V3 loop peptides), and biotinylated (V3_HxBc2_ and V3_BAL_), respectively. Fluorescein was introduced by coupling two eq. fluorescein-*N*-hydroxysuccinimide/5% DIPEA in DMF in the dark overnight, and biotin by coupling three eq. of biotin/DIC/HOBt overnight.

Peptides were cleaved from the resin using Reagent K (TFA/water/phenol/thioanisole/1,2-ethanedithiol; 82.5:5:5:2.5), precipitated in a cold 1:1 mixture of cyclohexane and tert-butyl methyl ether, extracted with water, lyophilized twice, and purified by preparative HPLC (conditions: column: Dr. Maisch Reprosil 100, 250 mm × 25 mm, flow rate: 9 mL/min, gradient: 30–60% (CX4-M1 and variants) and 15–45% (V3 loop peptides) acetonitrile in H_2_O (both containing 0.1% TFA) in 60 min and UV detection at 216 and 280 nm). Peptides were cyclized by air oxidation at 0.3 mg/mL in 50% acetonitrile in 0.1 M ammonium acetate, pH 8, for 3 days. Absence of free SH groups was confirmed by a negative Ellman’s test (18). Full length V3 loop peptides were characterized by MALDI-TOF mass spectrometry (MS) using MTP 384 massive target, Autoflex 1 (Bruker Daltonics), and FlexAnalysis Software (Bruker Daltonics). CX4-M1 and its variants, as well as truncated V3 loop peptides were characterized by analytical HPLC with online ESI-MS detection (LC-MS). Conditions: column: Phenomenex Kinetex 2.6 μM C18 100Å, 50 mm × 2.1 mm, flow rate: 0.4 mL/min, gradient: 5–95% acetonitrile in H_2_O (both containing 0.1% TFA) in 15 min. MS data of all peptides are listed in Tables 1 and 2. Stock solutions of purified peptides were prepared at 1 mM in 50% acetonitrile/H_2_O.

**Table 1:** **MALDI-TOF mass spectrometry data of synthesized V3 loop peptides**.

Peptide	*M*_calc_ (g/mol)	[M + H]^+^	[M + 2H]^2+^
V3_IIIB_	4732.6	4733.5	2366.7
Fluo-V3_HxBc2_	4760.6	4762.3	2381.0
Bio-V3_HxBc2_	4628.5	4628.7	2315.2
V3_MN_	4724.4	4724.0	2362.0
Fluo-V3_BAL_	4503.1	4504.1	2252.5
Bio-V3_BAL_	4371.0	4371.8	2185.8
V3_ADA_	4537.1	4538.1	2269.4
V3_HxBc2_ R306A	4675.4	4676.1	2338.5
V3_HxBc2_ I307A	4718.4	4719.2	2360.0
V3_HxBc2_ R308A	4675.4	4676.3	2338.8
V3_HxBc2_ I309A	4718.4	4719.4	2360.5
V3_HxBc2_ Q310A	4703.4	4707.6	2352.9
V3_HxBc2_ R311A	4675.4	4676.0	2338.7
V3_HxBc2_ G312A	4774.5	4775.1	2388.4
V3_HxBc2_ P313A	4734.5	4734.8	2367.9
V3_HxBc2_ G314A	4774.5	4774.9	2387.5
V3_HxBc2_ R315A	4675.4	4675.8	2338.4
V3_HxBc2_ F317A	4684.4	4684.8	2342.9
V3_HxBc2_ V318A	4732.4	4732.6	2366.2
V3_HxBc2_ T319A	4730.5	4730.7	2365.8
V3_HxBc2_ I320A	4718.4	4718.3	2359.6
V3_HxBc2_ G321A	4774.5	4774.4	2388.0
V3_HxBc2_ K322A	4703.4	4707.0	2353.3
V3_HxBc2_ I323A	4718.4	4722.2	2360.8
V3_HxBc2_ G324A	4774.5	4777.6	2387.6
V3_HxBc2_ N325A	4717.5	4721.2	2360.3
V3_HxBc2_ M326A	4700.4	4703.7	2351.5

**Table 2:** **ESI-mass spectrometry data of synthesized peptides**.

Peptide	*M*_calc_ (g/mol)	[M + H]^+^	[M + 2H]^2+^	[M + 3H]^3+^	[M + 4H]^4+^	[M + 5H]^5+^	[M + 6H]^6+^	[M + 7H]^7+^	[M + 8H]^8+^
CX4-M1	7136.3				1785.7	1428.5	1190.7	1020.8	893.1
D182A	7092.4				1773.6	1419.8	1183.2	1014.1	887.5
R183A	7050.1				1763.9	1410.5	1175.7		
Y184A	7044.5				1762.5	1410.0	1174.9	1007.1	881.7
I185A	7094.2				1773.9	1419.8	1183.3	1014.4	887.8
D187A	7092.4					1418.8	1182.2	1013.6	
R188A	7050.1					1410.6	1175.7	1007.8	
F189A	7060.3				1765.9	1413.1	1177.6	1009.9	883.8
Y190A	7044.5				1762.1	1410.0	1174.9	1007.4	881.6
P191A	7110.3				1778.8	1422.9	1186.1	1016.9	890.1
N192A	7093.3				1774.5	1419.3	1183.2	1014.2	887.7
D193A	7092.4					1418.9	1182.7	1014.0	
L194A	7094.3				1774.8	1419.8	1183.3	1014.5	887.8
W195A	7021.2				1756.3	1405.0	1171.2	1004.1	878.6
V196A	7108.3				1778.1	1422.5	1185.5	1016.2	889.7
V3_HxBc2_ turn	1585.7	1588.0	794.6	530.4	397.7				
V3_HxBc2_ turn/beta	2585.0		1292.7	862.5	647.4	517.8			
V3_HxBc2_ Δbeta	3761.2			1255.0	941.7	754.0	628.4	539.0	471.3

### Direct ELISA

High binding microtiter plates (Immulon 2HB) were coated overnight at 4°C with streptavidin (4 μg/mL) in 0.1 M sodium carbonate buffer pH 9.5. Unspecific binding was blocked with 1% BSA in 0.1 M phosphate buffer pH 7.2, 200 μL/well for 1 h. All following steps were performed using 0.1 M phosphate buffer pH 7.2 containing 1% BSA (ELISA with V3 loop peptides) and 0.1% BSA (ELISA with gp120), respectively, as well as 0.01% Tween 20. Plates were incubated with 100 μL CX4-M1 variants (0.5 μM for binding to V3 loop peptides, 2.5 μM for binding to gp120), or biotin alone as a blank, for 2 h. Plates were then incubated for 3 h with fluoresceinylated V3 loop peptides (250 or 100 nM), or gp120 (8.3 nM, either with or without 8.3 nM sCD4). V3 loop peptides were detected using 100 μL/well mouse anti-fluorescein-HRP conjugate from antibodies online (3 μg/mL) for 1 h. Gp120 was detected using a two antibody system. Sheep anti-gp120 (D7324) from Aalto Bio Reagents (0.2 μg/mL) was added for 1 h, followed by a 1 h incubation with rabbit anti-sheep-HRP conjugate from Dianova (3 μg/mL), 100 μL/well. Plates were washed four times with 0.01% Tween 20 in 0.1 M phosphate buffer pH 7.2 (300 μL/well) after each incubation step. Plates were developed with 100 μL/well OPD (1 mg/mL) in the presence of 0.03% H_2_O_2_ for approximately 1.5 min (V3 loop peptide detection), or 3.5 min (gp120 detection) in the dark. After the reaction was stopped with 50 μL/well 2 M H_2_SO_4_, absorbances were read at 492 nm and corrected for the blank (sample with biotin instead of CX4-M1). All data points present means of at least duplicates.

### Anti-V3 mAb ELISA

A streptavidin-coated plate, which was prepared as described above, was incubated with biotinylated V3 loop peptides at 100 nM for 2 h. Additionally, a high binding microtiter plate (Immulon 2HB) was coated with gp120 at 8 nM overnight, followed by blocking with 1% BSA. Both plates were incubated with anti-V3 loop mAbs (3869 or 447-52D), respectively, at 2 nM for 3 h. Bound antibody was detected using goat anti human HRP conjugate from Sigma-Aldrich at 1 μg/mL. Plates were developed as described above.

### Competitive ELISA

High binding microtiter plates (Immulon 2HB) were coated with streptavidin and incubated with CX4-M1 as described above for the direct ELISA. The anti-V3 loop mAbs 3869 and 447-52D, respectively, were added at twofold serial dilutions from 150 to 0.3 nM. Plates were then incubated for 3 h with gp120_HxBc2_ and sCD4 (each at 12.5 nM), or with V3_HxBc2_ (50 nM). Fluoresceinylated V3 loop peptides and gp120 were detected and plates developed as described above. IC_50_-values were determined using the regression wizard of Sigma Plot 9.0. Inhibition was calculated according to the following formula:

$$\%\;\mathrm{Inhibition}\;=\;\left[1\;-\;\left(A_{\text{antibody}}\;-\;A_{\text{blank}}\right)∕\left(A_{100\%}\;-\;A_{\text{blank}}\right)\right]\times 100$$

in which “100%” is a sample with CX4-M1, but without antibody, and “blank” is a sample with biotin instead of CX4-M1, and without antibody.

### Surface plasmon resonance

All measurements were performed using a Biacore X100 instrument in conjunction with the Biotin CAPture Kit (both from GE Healthcare). Sensor chip CAP was hybridized with a 1:10 dilution of CAPture reagent (streptavidin conjugate) in running buffer. CX4-M1 (2 μM) was bound to streptavidin and binding of V3 loop peptides (5–0.3 μM) or gp120 (8–0.5 nM) to CX4-M1 was measured. For dehybridization, a regeneration solution of 6 M guanidinium chloride and 250 mM sodium hydroxide was used. HBS-N buffer (10 mM HEPES, 150 mM sodium chloride, pH 7.4) containing 0.05% Tween 20, as well as 100 mM (V3 loop peptides) or 2.5 mM (gp120) magnesium chloride, was used as running buffer. Measurement and data evaluation was performed using Biacore X100 Control Software (Version 2.0) and Biacore X100 Evaluation Software (Version 2.0), respectively.

### Reporter cell assay

The indicator cell line CEMx174, which was kindly provided by Means and Desrosiers, contained the gene for the secreted alkaline phosphatase (SEAP) under the control of the simian immunodeficiency virus long terminal repeat (19). The suitability of this cell line for HIV-1 drug resistance testing has been evaluated extensively (20). Peptides were added to the cells prior to addition of HIV-1_NL4-3_. Three days after infection, cell culture supernatants were removed and analyzed for SEAP activity using the Phospha-Light kit (Life Technologies, Darmstadt, Germany) according to the manufacturer’s instructions. Data represent mean and standard error of at least three independently performed experiments, which were all carried out in triplicates.

## Results and Discussion

**Figure 2:**
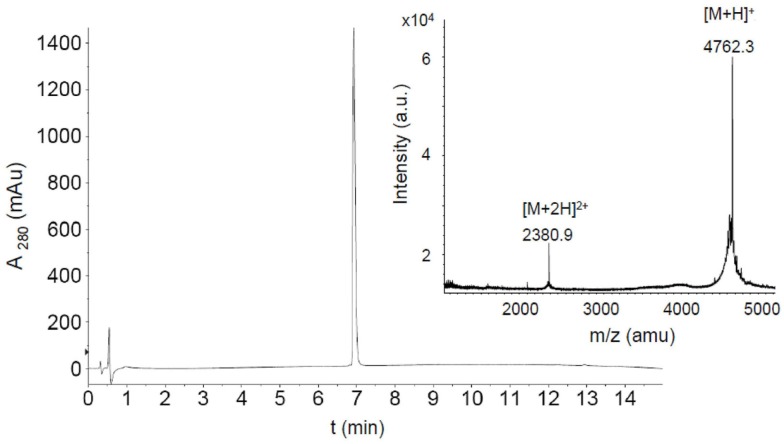
**HPLC chromatogram and MALDI-TOF mass spectrum of purified fluoresceinylated V3_HxBc2_**. (*M* = 4760.6).

**Figure 3:**
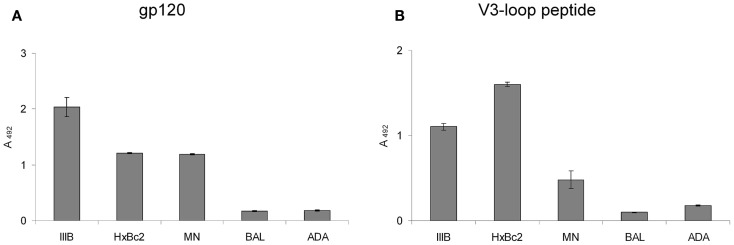
**Binding of gp120 (in the presence of sCD4) (A) and V3 loop peptides (B) from X4 tropic (IIIB, HxBc2, and MN) and R5-tropic (BAL and ADA) HIV-1 to the CXCR4 mimetic peptide CX4-M1**. See Section “Materials and Methods” (direct ELISA) for experimental detail. Error bars present deviations from the mean.

We have recently shown that the CXCR4 mimetic peptide CX4-M1 selectively binds to gp120 from X4 tropic HIV-1 (15). Since the coreceptor tropism of HIV-1 is largely located in the V3 loop of gp120 (21), we now asked the question whether the X4-selective interaction of CX4-M1 with gp120 could be reproduced in its interaction with peptides presenting the V3 loops of X4-tropic gp120. In order to answer this question, we prepared peptides presenting the V3 loops of gp120 from three X4-tropic (IIIB, HxBc2, MN) and two R5-tropic (BAL and ADA) HIV-1 strains (Figure 2; Table 3). These peptides were tested in a direct ELISA for their ability to bind to CX4-M1 (Figure 3). CX4-M1 recognized the V3 loop peptides (Figure 3B) with a selectivity similar to that seen for its interaction with gp120 (Figure 3A), i.e., V3 loop peptides derived from X4-tropic HIV-1 were bound much stronger than the respective R5-peptides.

**Table 3:** **Sequences of V3 loop peptides**. 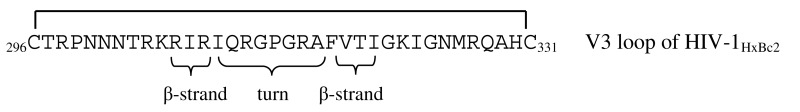

Peptide	Sequence
**V3 LOOP PEPTIDES (WT)**	
V3_IIIB_	Fluo^a^-X^b^-G-X-CTRPNNNTRKKIRIQRGPGRAFVTIGKIGNMRQAHC-NH_2_
V3_HxBc2_	Yc-X-G-X-CTRPNNNTRKRIRIQRGPGRAFVTIGKIGNMRQAHC-NH_2_
V3_MN_	Fluo-X-G-X-CTRPNYNKRKRIHI–GPGRAFYTTKNIIGTIRQAHC-NH_2_
V3_BAL_	Yc-X-G-X-CTRPNNNTRKSIHI–GPGRALYTTGEIIGDIRQAHC-NH_2_
V3_ADA_	Fluo-X-G-X-CTRPNNNTRKSIHI–GPGRAFYTTGEIIGDIRQAHC-NH_2_
**TRUNCATED VARIANTS of V3_HxBc2_**	
V3_HxBc2_ turn	Fluo-X-G-X––––––-C^d^QRGPGRAC^d^–––––––-NH_2_
V3_HxBc2_ turn/beta	Fluo-X-G-X––––-C^d^RIRIQRGPGRAFVTIC^d^–––––-NH_2_
V3_HxBc2_ Δbeta	Fluo-X-G-X-CTRPNNNTRK––QRGPGRA–-GKIGNMRQAHC–NH_2_
**ALANINE SCAN of V3_HxBc2_**	
V3_HxBc2_ R306A	Fluo-X-G-X-CTRPNNNTRKAIRIQRGPGRAFVTIGKIGNMRQAHC-NH_2_
V3_HxBc2_ I307A	Fluo-X-G-X-CTRPNNNTRKRARIQRGPGRAFVTIGKIGNMRQAHC-NH_2_
V3_HxBc2_ R308A	Fluo-X-G-X-CTRPNNNTRKRIAIQRGPGRAFVTIGKIGNMRQAHC-NH_2_
V3_HxBc2_ I309A	Fluo-X-G-X-CTRPNNNTRKRIRAQRGPGRAFVTIGKIGNMRQAHC-NH_2_
V3_HxBc2_ Q310A	Fluo-X-G-X-CTRPNNNTRKRIRIARGPGRAFVTIGKIGNMRQAHC-NH_2_
V3_HxBc2_ R311A	Fluo-X-G-X-CTRPNNNTRKRIRIQAGPGRAFVTIGKIGNMRQAHC-NH_2_
V3_HxBc2_ G312A	Fluo-X-G-X-CTRPNNNTRKRIRIQRAPGRAFVTIGKIGNMRQAHC-NH_2_
V3_HxBc2_ P313A	Fluo-X-G-X-CTRPNNNTRKRIRIQRGAGRAFVTIGKIGNMRQAHC-NH_2_
V3_HxBc2_ G314A	Fluo-X-G-X-CTRPNNNTRKRIRIQRGPARAFVTIGKIGNMRQAHC-NH_2_
V3_HxBc2_ R315A	Fluo-X-G-X-CTRPNNNTRKRIRIQRGPGAAFVTIGKIGNMRQAHC-NH_2_
V3_HxBc2_ F317A	Fluo-X-G-X-CTRPNNNTRKRIRIQRGPGRAAVTIGKIGNMRQAHC-NH_2_
V3_HxBc2_ V318A	Fluo-X-G-X-CTRPNNNTRKRIRIQRGPGRAFATIGKIGNMRQAHC-NH_2_
V3_HxBc2_ T319A	Fluo-X-G-X-CTRPNNNTRKRIRIQRGPGRAFVAIGKIGNMRQAHC-NH_2_
V3_HxBc2_ I320A	Fluo-X-G-X-CTRPNNNTRKRIRIQRGPGRAFVTAGKIGNMRQAHC-NH_2_
V3_HxBc2_ G321A	Fluo-X-G-X-CTRPNNNTRKRIRIQRGPGRAFVTIAKIGNMRQAHC-NH_2_
V3_HxBc2_ K322A	Fluo-X-G-X-CTRPNNNTRKRIRIQRGPGRAFVTIGAIGNMRQAHC-NH_2_
V3_HxBc2_ I323A	Fluo-X-G-X-CTRPNNNTRKRIRIQRGPGRAFVTIGKAGNMRQAHC-NH_2_
V3_HxBc2_ G324A	Fluo-X-G-X-CTRPNNNTRKRIRIQRGPGRAFVTIGKIANMRQAHC-NH_2_
V3_HxBc2_ N325A	Fluo-X-G-X-CTRPNNNTRKRIRIQRGPGRAFVTIGKIGAMRQAHC-NH_2_
V3_HxBc2_ M326A	Fluo-X-G-X-CTRPNNNTRKRIRIQRGPGRAFVTIGKIGNARQAHC-NH_2_

^a^Fluo, fluorescein; ^b^X, ε-aminohexanoic acid (spacer); ^c^Y, fluorescein or biotin; ^d^additional cysteine residues forming a disulfide bridge.

**Figure 4:**
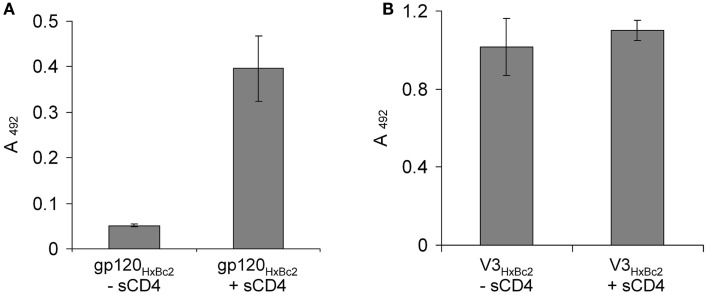
**Binding of the CXCR4 mimetic peptide CX4-M1 to gp120_HxBc2_ (A) and the V3 loop peptide V3_HxBc2_ (B), in the presence and absence, respectively, of sCD4**. See Section “Materials and Methods” (direct ELISA) for experimental detail. Error bars present deviations from the mean.

During HIV-1 entry into cells, the interaction of HIV-1 gp120 and coreceptor is contingent on prior contact with CD4, which opens up the gp120 conformation, exposing its coreceptor binding site, which includes the V3 loop (11). Likewise, binding of gp120 to CX4-M1 can be enhanced by soluble CD4 (sCD4) (15). The interaction of X4-V3 loop peptides with CX4-M1, on the other hand, should be independent on the presence of sCD4, since in this interaction the V3 loop is not conformationally masked within gp120, i.e., it is exposed right from the start. This could be experimentally confirmed in binding experiments in the presence and absence of sCD4, which clearly showed that, unlike its interaction with gp120, binding of CX4-M1 to V3 loop peptides does not need enhancement by sCD4 (Figure 4).

**Figure 5:**
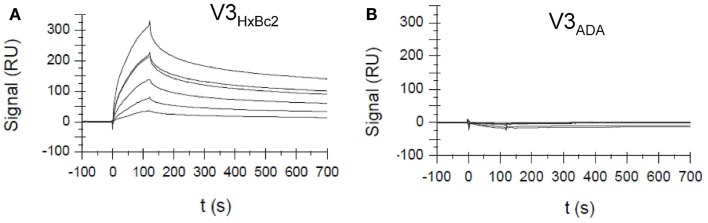
**Surface Plasmon resonance sensograms for the interaction of the CXCR4 mimetic peptide CX4-M1 with V3 loop peptides from X4 tropic (HxBc2) (A) and R5-tropic (ADA) (B) HIV-1**. See Section “Materials and Methods” for experimental detail.

Using surface plasmon resonance measurements, we could show that CX4-M1 binds to gp120 from X4 tropic HIV-1 strains, as well as to the respective V3 loop peptides, with *K*_D_ values in the low to submicromolar range (Figure 5; Table 4). On the other hand, no binding was detected to gp120, or to the respective V3 loop peptides, from R5-tropic HIV-1 strains. Apart from re-confirming the tropism selectivity of the CX4-M1–gp120 interaction, these results also indicate that HIV-1 coreceptor tropism can be reproduced in V3 loop peptides. It should be noted, however, that, unlike in the ELISA experiments described above, no sCD4 was present in these SPR experiments. As shown before (Figure 4), sCD4 dramatically enhances the interaction of gp120_HxBc2_ with CX4-M1, likely by inducing proper exposure of the V3 loop. This phenomenon is apparently less pronounced for gp120_IIIB_ than for gp120_HxBc2_, since the former protein has an approximately 10-fold higher affinity to CX4-M1 than the respective V3 loop peptide, even when no sCD4 is present (Table 4). Regardless of these differences, it is likely that, in the presence of sCD4, the *K*_D_ values for the X4 tropic gp120–CX4-M1 interactions would be considerably lower than those for the respective V3 loop peptide interactions.

**Table 4:** **Affinities of CX4-M1 to gp120 and V3 loop peptides, respectively, from X4 tropic and R5-tropic HIV-1**.

HIV-1 strain (tropism)	*K*_D_ (gp120)	*K*_D_ (V3 loop peptide)
IIIB (X4)	0.1 μM	1.1 μM
HxBc2 (X4)	0.2 μM	0.3 μM
MN (X4)	0.7 μM	1.6 μM
ADA (R5)	No binding	No binding
BAL (R5)	No binding	No binding

**Figure 6:**
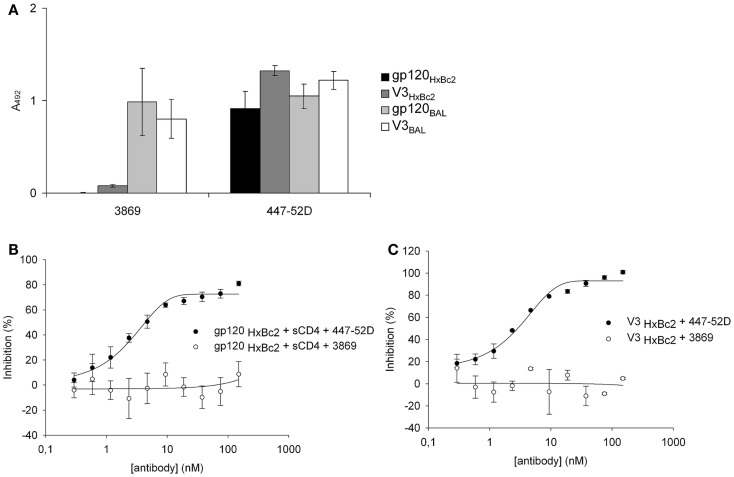
**Binding of gp120 and V3 loop peptides from HIV-1 strains HxBc2 (+QR) and BAL (−QR), to mAbs 3869 and 447-52D (A)**. Dose-dependent effect of mAbs 447-52D and 3869, respectively, on binding of gp120_HxBc2_ (in the presence of sCD4) **(B)** and the respective V3 loop peptide (V3_HxBc2_) **(C)**, to the CXCR4 mimetic peptide CX4-M1. See Section “Materials and Methods” (anti-V3 mAb ELISA and Competitive ELISA) for experimental detail. Error bars present deviations from the mean.

The sequences of the gp120 V3 loops of very few HIV-1 strains, including IIIB and HxBc2, contain an additional QR dipeptide, which is inserted in the central region of the sequence (see Table 3, first two entries). The presence or absence of this QR insert (+QR vs. −QR) is irrelevant for coreceptor tropism, however, we have found that it is reflected in the binding selectivity of antibodies that recognize the V3 loop. MAb 447-52D, for example, recognizes both +QR and −QR V3 loops, such as HxBc2 and BAL, whereas mAb 3869 is selective for −QR V3 loops (Figure 6A). We have used these two antibodies to further substantiate the mimicry of binding selectivity of gp120 in its interaction with CX4-M1, by V3 loop peptides. In competition experiments we could show that mAb 447-52D, but not mAb 3869, is able to compete with CX4-M1 for binding to gp120_HxBc2_ (Figure 6B), as well as to the respective V3 loop peptide (Figure 6C). These results provide further evidence for selectivity in binding of not only gp120, but also V3 loop peptides, to the CXCR4 mimetic peptide CX4-M1, and thus for a functional mimicry of the gp120–CXCR4 interaction, by the V3 loop–CX4-M1 interaction.

**Figure 7:**
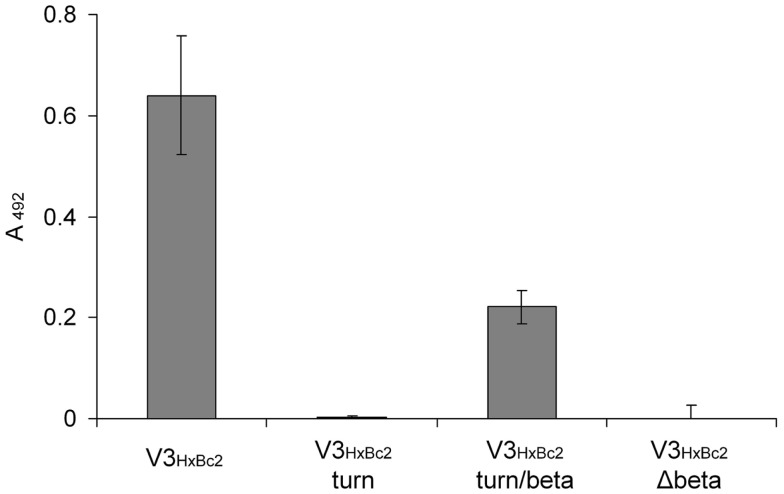
**Binding of V3_HxBc2_ and its truncated variants to the CXCR4 mimetic peptide CX4-M1**. See Section “Materials and Methods” (direct ELISA) for experimental detail. Error bars present deviations from the mean.

Having established the V3 loop–CX4-M1 interaction as a valid model for the gp120–CXCR4 interaction, we set out to characterize it at the level of individual amino acids. While peptides presenting the V3 loop are intrinsically unfolded (22–24), the sequence stretch covering residues 306–320 of gp120, which is located in the central region at the tip of the V3 loop, adopts a defined secondary structure in an antibody-bound stage. This was shown by NMR spectroscopy and X-ray crystallography of several complexes of V3 loop peptides with antibodies whose epitopes were mapped to the V3 loop (25–34). In these complex structures, residues 306–309, as well as 317–320, form strands of a small beta sheet, which are connected by the turn region located in amino acids 310–316 (HxBc2 nomenclature). In order to dissect the contribution of these V3 loop fragments to the interaction with CX4-M1, truncated variants of V3_HxBc2_ were generated, which present only the turn region (V3_HxBc2_ turn), or the turn region and the two beta strands (V3_HxBc2_ turn/beta), as well as a peptide in which the two beta strands were omitted (V3_HxBc2_ Δbeta). In order to facilitate a possible formation of the turn and beta sheet, the sequences of V3_HxBc2_ turn and V3_HxBc2_ turn/beta were flanked by additional cysteine residues, which were connected by disulfide bridges, generating covalently stabilized loops. Of the three truncated V3_HxBc2_ variants, only V3_HxBc2_ turn/beta retained some of the affinity of V3_HxBc2_ to CX4-M1 (Figure 7), suggesting a contribution of the beta strands to the interaction with CX4-M1, since their omission in peptides V3_HxBc2_ turn and V3_HxBc2_ Δbeta resulted in a complete loss of binding to CX4-M1.

**Figure 8:**
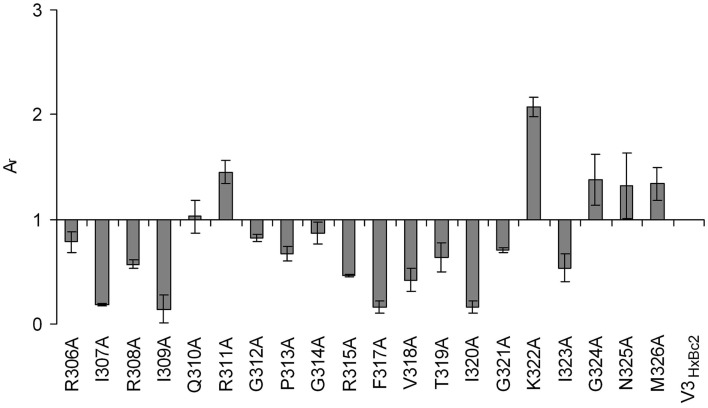
**Effect of alanine substitutions in V3_HxBc2_ on binding to the CXCR4 mimetic peptide CX4-M1**. See Section “Materials and Methods” (direct ELISA) for experimental detail. Relative absorbances (*A*_r_) were calculated according to the following formula: *A*_r_ = (*A*_peptide_ − *A*_blank_)/(*A*_c_ − *A*_blank_), in which “peptide” refers to the respective variant of V3_HxBc2_, and “c” refers to V3_HxBc2_. Error bars present deviations from the mean.

The importance of the two beta strand fragments for the interaction with CX4-M1 could be confirmed using peptides presenting an alanine scan of residues 306–326 of V3_HxBc2_ (Table 3). This segment presents the complete V3 loop except for the N- and C-termini (residues 296–305 and 327–331, respectively), which are conserved, regardless of coreceptor tropism (35). Similar to the truncated V3_HxBc2_ variants, binding data obtained for these peptides point to a strong contribution of the beta strands to the interaction of V3_HxBc2_ with CX4-M1, since replacement of these residues, in particular I307, R308, I309, F317, V318, and I320, with alanine, strongly diminished binding to CX4-M1 (Figure 8). This result may suggest that the beta sheet in the V3 loop peptides, which is seen in V3 loop-antibody complexes, could be reproduced in the CX4-M1 – bound state of the V3 loop peptides, and that this beta sheet may possibly be affected by modifying the amino acids constituting the beta strands.

Since the second extracellular loop (ECL2) of CXCR4 is crucial for the interaction of this coreceptor with HIV-1 gp120 (36), we set out to dissect the determinants of this loop, at the level of individual amino acids, using CX4-M1 as a CXCR4 mimic. Binding and infection assays involving cells that express CXCR4 containing alanine mutations in ECL2 had previously indicated that replacing acidic residues D187 and D193 with alanine had an inhibitory effect (37). These effects were further confirmed in double mutants, in which an aspartate (D187) and a tyrosine residue (Y190) were both replaced by alanine (38). Furthermore, a charge dependency in the interaction of the V3 loop with CXCR4 is highly likely, since the X4-tropism of V3 loops strongly depends on the presence of positively charged amino acids in positions 306, 321, and/or 322 (HxBc2 nomenclature) (39).

**Figure 9:**
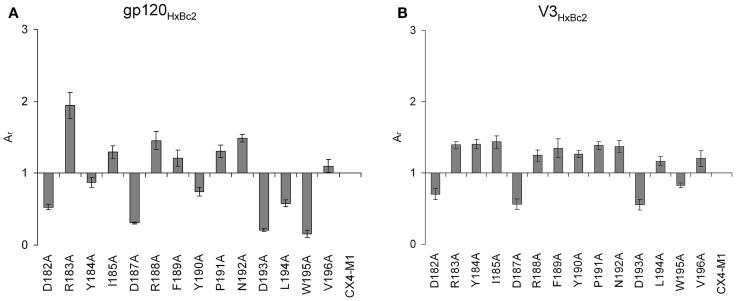
**Effect of alanine substitutions in ECL2 of CX4-M1 on binding to gp120_HxBc2_ (A) (in the presence of sCD4) and its corresponding V3 loop peptide (V3_HxBc2_) (B), respectively**. See Section “Materials and Methods” (direct ELISA) for experimental detail. Relative absorbances (*A*_r_) were calculated according to the following formula: *A*_r_ = (*A*_peptide_ − *A*_blank_)/(*A*_c_ − *A*_blank_), in which “peptide” refers to the respective variant of CX4-M1, and “c” refers to CX4-M1. Error bars present deviations from the mean.

Using an alanine scan of ECL2 in the context of CX4-M1 (Table 5), we identified positively charged ECL2 residues, such as R183 and R188, but also I185, F189, P191, and N192, whose replacement with alanine enhanced binding to gp120 from X4 tropic HIV-1_HxBc2_ (Figure 9A). Replacement of other ECL2 residues (D182, D187, D193, L194A, W195) with alanine, on the other hand, had a negative effect on the affinity, indicating their importance for the CX4-M1–gp120 interaction (Figure 9A). Interestingly, a similar, yet not identical pattern could be seen for the interaction of the ECL2 alanine scan of CX4-M1 with the V3 loop of gp120_HxBc2_ (Figure 9B). The inhibitory effect on binding of decreasing the negative net charge of CX4-M1, by replacing one of the three acidic residues of ECL2 (D182, D187, and D193) with alanine, was clearly seen also in its interaction with the V3_HxBc2_. On the other hand, enhancement of binding to V3_HxBc2_ was seen not only in R183A, R188A, I1854, F189, P191A, and N192A, but also in CX4-M1 variants with alanine mutations at aromatic residues (Y184 and Y190), as well as hydrophobic residues L194 and V196. Moreover, we detected a converse behavior of CX4-M1 variants Y184A, Y190A, and L194A, with attenuated binding to gp120, while binding to the V3 loop peptide was enhanced. These differences may have their origin in the different flexibility of the V3 loop in gp120 and the V3 loop peptide, respectively, in that the larger flexibility of the V3 loop peptide may be able to compensate for the complementarity constraints imposed by alanine mutations in CX4-M1. Furthermore, post-translational modifications in the V3 loop of gp120, such as *N*-linked glycosylation of asparagine residues (40), may also be a factor contributing to the differences in binding of some CX4-M1 variants to gp120 and the respective V3 loop peptide. In addition, the V3 loop is not the only part of gp120 that interacts with the coreceptor. Other gp120 regions, including the bridging sheet and the V1/V2 loop, also play a role in this interaction, even when the N-terminus of CXCR4, which is thought to bind to the bridging sheet, is deleted (41). Since the V1/V2 loop is not present in V3 loop peptides, the different binding pattern of CX4-M1 alanine variants to gp120 and V3 loop peptides may indicate interactions of the V1/V2 loop also with ECL2 of CXCR4.

**Table 5:** **Alanine substitution variants of the CXCR4 mimetic peptide CX4-M1**. 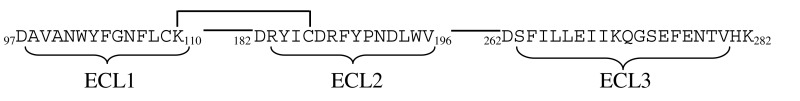

Peptide	Sequence
CX4-M1	Ac^a^-ECL1-X^b^-B^c^-X-ECL2-X-B-X-ECL3-X-K(Bio^d^)-NH_2_
**Alanine substitution variants of ECL2 of CX4-M1**
CX4-M1 D182A	Ac-ECL1-X-B-X-ARYICDRFYPNDLWV-X-B-X-ECL3-X-K(Bio)-NH_2_
CX4-M1 R183A	Ac-ECL1-X-B-X-DAYICDRFYPNDLWV-X-B-X-ECL3-X-K(Bio)-NH_2_
CX4-M1 Y184A	Ac-ECL1-X-B-X-DRAICDRFYPNDLWV-X-B-X-ECL3-X-K(Bio)-NH_2_
CX4-M1 I185A	Ac-ECL1-X-B-X-DRYACDRFYPNDLWV-X-B-X-ECL3-X-K(Bio)-NH_2_
CX4-M1 D187A	Ac-ECL1-X-B-X-DRYICARFYPNDLWV-X-B-X-ECL3-X-K(Bio)-NH_2_
CX4-M1 R188A	Ac-ECL1-X-B-X-DRYICDAFYPNDLWV-X-B-X-ECL3-X-K(Bio)-NH_2_
CX4-M1 F189A	Ac-ECL1-X-B-X-DRYICDRAYPNDLWV-X-B-X-ECL3-X-K(Bio)-NH_2_
CX4-M1 Y190A	Ac-ECL1-X-B-X-DRYICDRFAPNDLWV-X-B-X-ECL3-X-K(Bio)-NH_2_
CX4-M1 P191A	Ac-ECL1-X-B-X-DRYICDRFYANDLWV-X-B-X-ECL3-X-K(Bio)-NH_2_
CX4-M1 N192A	Ac-ECL1-X-B-X-DRYICDRFYPADLWV-X-B-X-ECL3-X-K(Bio)-NH_2_
CX4-M1 D193A	Ac-ECL1-X-B-X-DRYICDRFYPNALWV-X-B-X-ECL3-X-K(Bio)-NH_2_
CX4-M1 L194A	Ac-ECL1-X-B-X-DRYICDRFYPNDAWV-X-B-X-ECL3-X-K(Bio)-NH_2_
CX4-M1 W195A	Ac-ECL1-X-B-X-DRYICDRFYPNDLAV-X-B-X-ECL3-X-K(Bio)-NH_2_
CX4-M1 V196A	Ac-ECL1-X-B-X-DRYICDRFYPNDLWA-X-B-X-ECL3-X-K(Bio)-NH_2_

^a^Ac, acetyl; ^b^X, ε-aminohexanoic acid; ^c^B, β-alanine; ^d^Bio, biotin.

Taken together, these data support, at the level of peptide–protein and peptide–peptide interactions, the notion of a negative net charge of ECL2 of CXCR4 being critical for its interaction with the positively charged V3 loop of gp120 from X4-tropic HIV-1. Furthermore, these data demonstrate how variants of mimetic peptides can be used to gain information on a protein–protein interaction when peptide–peptide interactions are looked at in the context of the respective peptide–protein interaction.

**Figure 10:**
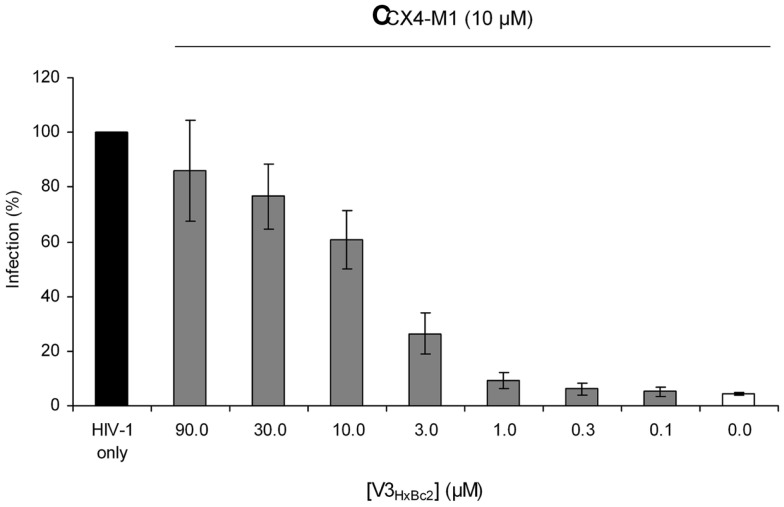
**Dose-dependent effect of the V3 loop peptide V3_HxBc2_ on the CX4-M1 – induced inhibition of infection of CEMx174 cells with HIV-1_NL4-3_**. See Section “Materials and Methods” (reporter cell assay) for experimental detail. Error bars present standard errors of the mean (SEM).

We have previously shown that CX4-M1 not only selectively binds to X4 tropic gp120, but also selectively inhibits infection of cells with X4-tropic HIV-1 (15). This inhibition is thought to be based on the interaction of CX4-M1 with viral gp120, which precludes contact of the virus with cellular CXCR4 and, consequently, intercepts the process of HIV-1 entry. Therefore, we tested V3_HxBc2_ for its ability to interfere with the infection inhibition caused by CX4-M1. As shown in Figure 10, CX4-M1 inhibits, at 10 μM, the infection of SEAP cells with the X4 tropic HIV-1_NL-4.3_ by more than 90% (white bar). This almost complete inhibition could be counter-acted, in a dose-dependent manner, by V3_HxBc2_, restoring infection of cells with HIV-1 (gray bars). It seems likely that this effect is due to capture of CX4-M1 by the V3 loop peptide, preventing binding of CX4-M1 to viral gp120, and, consequently, resulting in recovery of infection. This result shows that the functional mimicry of the gp120–CXCR4 interaction by the V3 loop–CX4-M1 interaction is present not only in binding experiments involving solely proteins and peptides, but also in the context of an HIV-1 infection.

## Conclusion

In conclusion, using direct and competitive binding assays, as well as an HIV-1 infection assay, we have provided experimental evidence indicating that the interaction of the HIV-1 glycoprotein gp120 with its cellular coreceptor CXCR4 can be functionally mimicked by peptides presenting essential parts of the binding sites of the two proteins for each other, i.e., the V3 loop of gp120 and the three ECLs of CXCR4. Furthermore, truncated and substitution variants of the mimetic peptides were used to explore this interaction at the level of individual amino acids, the results of which may be relevant for the analysis of the gp120–CXCR4 interaction as well. Ongoing and future research will be aimed at elucidating the structural basis of this interaction, as well as on probing the concept of mimicking protein–protein interactions by peptide–peptide interactions in the context of other proteins.

## Conflict of Interest Statement

The authors declare that the research was conducted in the absence of any commercial or financial relationships that could be construed as a potential conflict of interest.
